# Fungal fruit body assemblages are tougher in harsh microclimates

**DOI:** 10.1038/s41598-022-05715-9

**Published:** 2022-01-31

**Authors:** Franz-Sebastian Krah, Jonas Hagge, Jasper Schreiber, Roland Brandl, Jörg Müller, Claus Bässler

**Affiliations:** 1 Faculty of Biological Sciences, Institute for Ecology, Evolution and Diversity, Conservation Biology, Goethe University Frankfurt, Frankfurt am Main, Germany; 2 Forest Nature Conservation, Northwest German Forest Research Institute, Hann. Münden, Germany; 3 Forest Nature Conservation, Georg‐August‐University Göttingen, Göttingen, Germany; 4 Department of Ecology, Philips University of Marburg, Marburg, Germany; 5 Department of Animal Ecology and Tropical Biology, University of Würzburg, Würzburg, Germany; 6 Bavarian Forest National Park, Grafenau, Germany

**Keywords:** Climate-change ecology, Community ecology, Conservation biology

## Abstract

Forest species are affected by macroclimate, however, the microclimatic variability can be more extreme and change through climate change. Fungal fruiting community composition was affected by microclimatic differences. Here we ask whether differences in the fruiting community can be explained by morphological traits of the fruit body, which may help endure harsh conditions. We used a dead wood experiment and macrofungal fruit body size, color, and toughness. We exposed logs of two host tree species under closed and experimentally opened forest canopies in a random-block design for four years and identified all visible fruit bodies of two fungal lineages (Basidio- and Ascomycota). We found a consistently higher proportion of tough-fleshed species in harsher microclimates under open canopies. Although significant, responses of community fruit body size and color lightness were inconsistent across lineages. We suggest the toughness-protection hypothesis, stating that tough-fleshed fruit bodies protect from microclimatic extremes by reducing dehydration. Our study suggests that the predicted increase of microclimatic harshness with climate change will likely decrease the presence of soft-fleshed fruit bodies. Whether harsh microclimates also affect the mycelium of macrofungi with different fruit body morphology would complement our findings and increase predictability under climate change.

## Introduction

Analyses of functional traits help to better understand and predict species community change in response to environmental change^1,2^. Organism body size, color, or toughness are important traits related to the thermal climate. Climate warming has already decreased body size and increased light-colored communities^3,4^. Besides the average annual temperature increase, forest organisms experience heterogeneous microclimatic conditions, e.g., because of canopy cover change^5,6^. Open forest stands are characterized by extreme temperatures and radiation^7–10^, which can even exceed macroclimatic mean differences^6,8^ (hereafter “harsh microclimate”). Closed canopies, in contrast, buffer these extremes^8,11^. Forest management activities, natural disturbances, and climate change increase canopy loss, leading to harsher microclimates^12–15^. Previous studies showed that many forest species groups differ in community composition between closed and open canopies^16,17^. Although fungal fruiting communities were previously found to differ between microclimates^17,18^, we currently do not know if fruit body traits differ as well.

Fungi are ectotherm, and modular organisms, characterized by a mycelium (consisting of hyphae), and many species produce multicellular fruit bodies (hereafter “macrofungi”) for sexual reproduction and the subsequent development of spores^19^. Mycelium grows within the substrate, e.g., soil or dead wood, exploiting resources (e.g., decay of organic matter)^20^. Before fruiting, the mycelium must reach a critical size (e.g., biomass) with a critical amount of storage mycelium, which serves fruit body production^21,22^. Before maturation, fruiting cues start the fructification process by forming fruiting body initials, primordia, maturating fruit bodies, and finally mature fruit bodies with sexual spores^22,23^. Species that can successfully grow as mycelium and form mature fruit bodies in a given environment must endure the below- and above-ground conditions. Both modules have evolved strategies to cope with stressful conditions^24,25^. The presence of a fruit body in an environment requires biochemical^25^ and morphological adaptations of the fruit body^26–29^. Several studies provided evidence that fruit body traits are under selection^30–32^ and can function to tolerate harsh climate conditions^33,34^. However, whether the presence of morphological traits of the fruit body is related to the microclimate is currently unknown. We thus expect non-random fruit body trait distribution with microclimates, which would imply morphological features of the fruit bodies that enhance their survival. The absence of fruit bodies, however, may be the result of multiple processes. First, the environment does not allow a species to grow as mycelium, and hence fruit bodies are also absent. Second, a species grows as mycelium within the environment, but fruit body production is absent (e.g., because a minimal storage-mycelium necessary to produce fruiting structures could not be formed^21^). Third, a species grows as mycelium within the environment, but production of fruit bodies is prohibited, e.g., absence of fruiting cues or physiological damage during growth of primordia, growth of fruit bodies, and after maturation.

We currently have little understanding of which fruit body traits may be related to harsh microclimates. Two macroecological studies demonstrated that fruit body size and color lightness are correlated with the climate on a large spatial scale^26,27^. European fruiting communities were darker in cold environments, possibly as an adaptation to increase fruit body temperature and thus improve spore production^26^. On a global scale, fruiting communities had smaller fruit bodies in hot or cold environments, possibly an adaptation for cooling down or heating more rapidly^27^. Further, one study tested the effect of forest edges (characterized by sun exposure) on fruit body morphology and found a higher richness of hard-fleshed, long-lived fruit bodies at the forest edge^29^. Taking these results together, fruit body morphology may allow the fruit body to endure harsh conditions, such as temperature variability or UV radiation^33,35^. However, whether morphological traits of the fruit body show pattern that might explain differences in the fruiting communities between microclimates has not been tested.

If fruit body size, color lightness, and toughness are related to harsh microclimate conditions as outlined above, the following responses of the fruiting community can be expected: (1) based on considerations of the surface-area-to-volume ratio, one might expect larger or smaller fruit bodies in harsh microclimates. Larger fruit bodies have higher thermal inertia, whereas smaller ones can get rid of excess heat faster (‘heat conservation hypothesis’^36^, and ‘heat-up-cool-down-hypothesis’^27^). Both may potentially lower heat stress under open canopies. (2) Based on considerations of the pigmentation of fruit bodies, one might expect more bright- or dark-colored fruit bodies in harsh microclimates. Strong pigmentation of fruit bodies may lower the damaging effect of radiation and desiccation under open canopies, because melanin pigmentation reduces oxidative stress (‘melanism-desiccation hypothesis’^37^ and ‘photo-protection hypothesis’^38^). Weak pigmentation may reduce heat stress, which increases with increasingly darker fruit bodies (‘thermal-melanism hypothesis’^26,39^). (3) Based on considerations of the toughness of fruit bodies, one might expect more tough-fleshed fruit bodies in harsh microclimates. The toughness of the fruit body may lower heat and desiccation stress by reducing the transpiration rate (‘toughness-protection hypothesis’). Here we thus test the pattern of community traits that might result from different processes described by these hypotheses.

**Figure 1:**
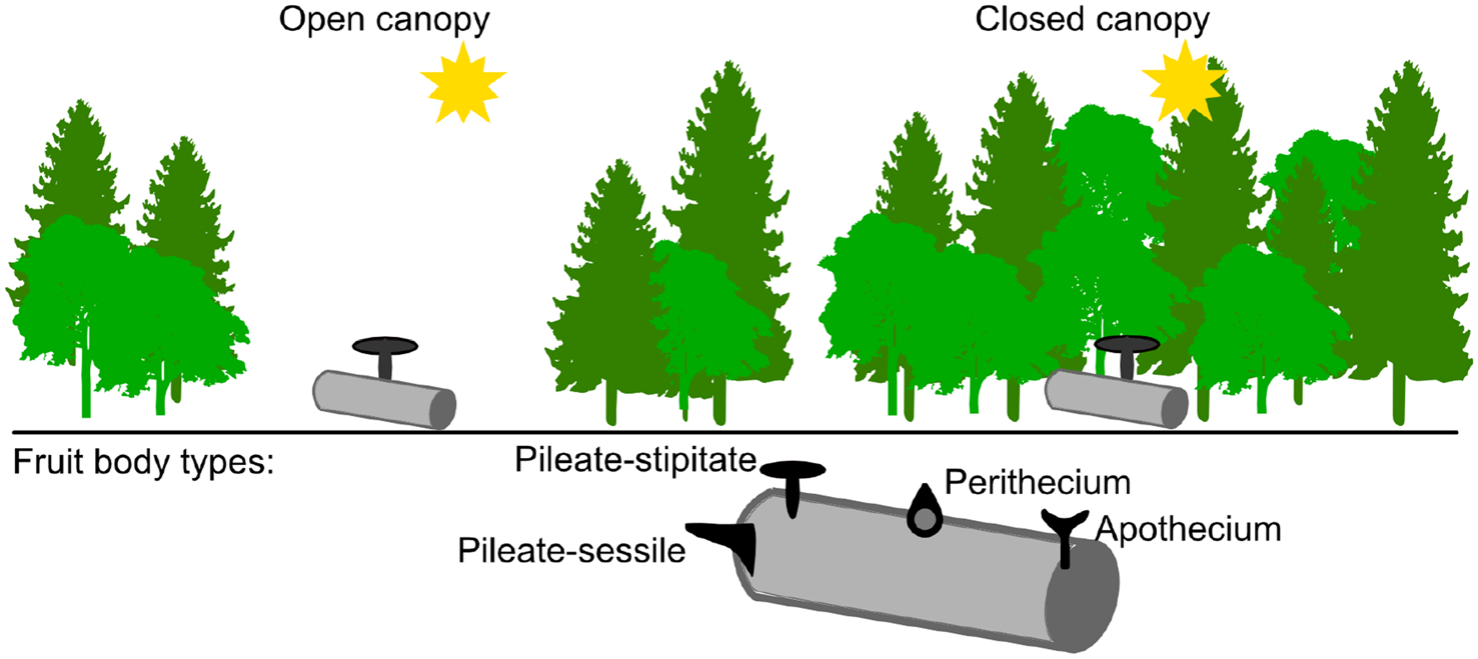
Conceptual representation of the study treatment and fruit body types. Random block design with five blocks, each with 12 plots with open and 12 with a closed canopy. Forest stands with open canopies are characterized by direct sun exposure and thus increased radiation, heat, and drought (“harsh microclimate”), compared with forest stands with closed canopies^7,11^. We investigated trait change of four fruit body types: pileate-stipitate and pileate-sessile for Basidiomycota and Perithecia and Apothecia for Ascomycota. Illustration by F.-S. Krah.

We used a dead-wood experiment on a landscape scale and manipulated dead-wood logs of two tree species under closed canopies and experimentally created forest gaps (0.1 ha, hereafter “open canopies”, Fig. 1). Fungal fruiting communities were assessed via fruit body-based inventories across four years of the initial phase of decomposition and 320 dead-wood logs of the same size. We assembled trait data of fruit body size from literature, fruit body color lightness from photographs, and classified fungi into soft- and tough-fleshed (toughness) based on expert knowledge. We calculated fungal fruiting community traits for presence/absence data, and we further applied a null model to test against a random distribution of species in the microclimates to approach our expectations. Here we address the overall question of whether fruit body size, color and toughness of communities are related to microclimate harshness.

## Results

**Figure 2:**
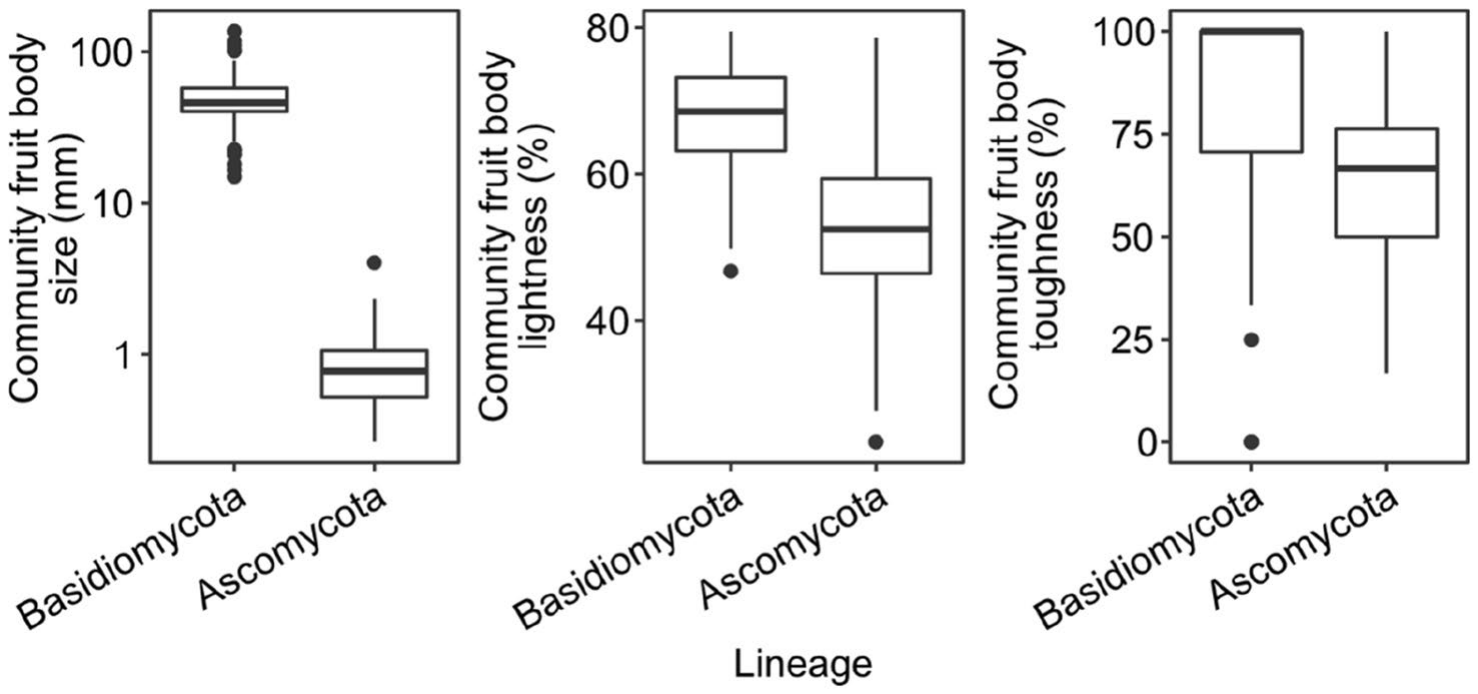
Differences in community fruit body traits between lineages. Note that fruit body size is log_10_-transformed. We used linear mixed-effects models with a nested random effect on block and plot to test for significance. Statistics values are given in the text. R programming environment version 4.1.2^40^ and R add-on package *ggplot2*^41^.

We found significantly larger (LME: z value = − 67.00, p < 0.001) and more tough-fleshed fruit bodies (LME: z value = − 5.11, p < 0.001) for Basidiomycota compared with Ascomycota (Fig. 2). Community fruit body lightness was also higher in Basidio- compared with Ascomycota, but not significantly (LME: z value = 1.73, p = 0.083, Fig. 2).

**Figure 3:**
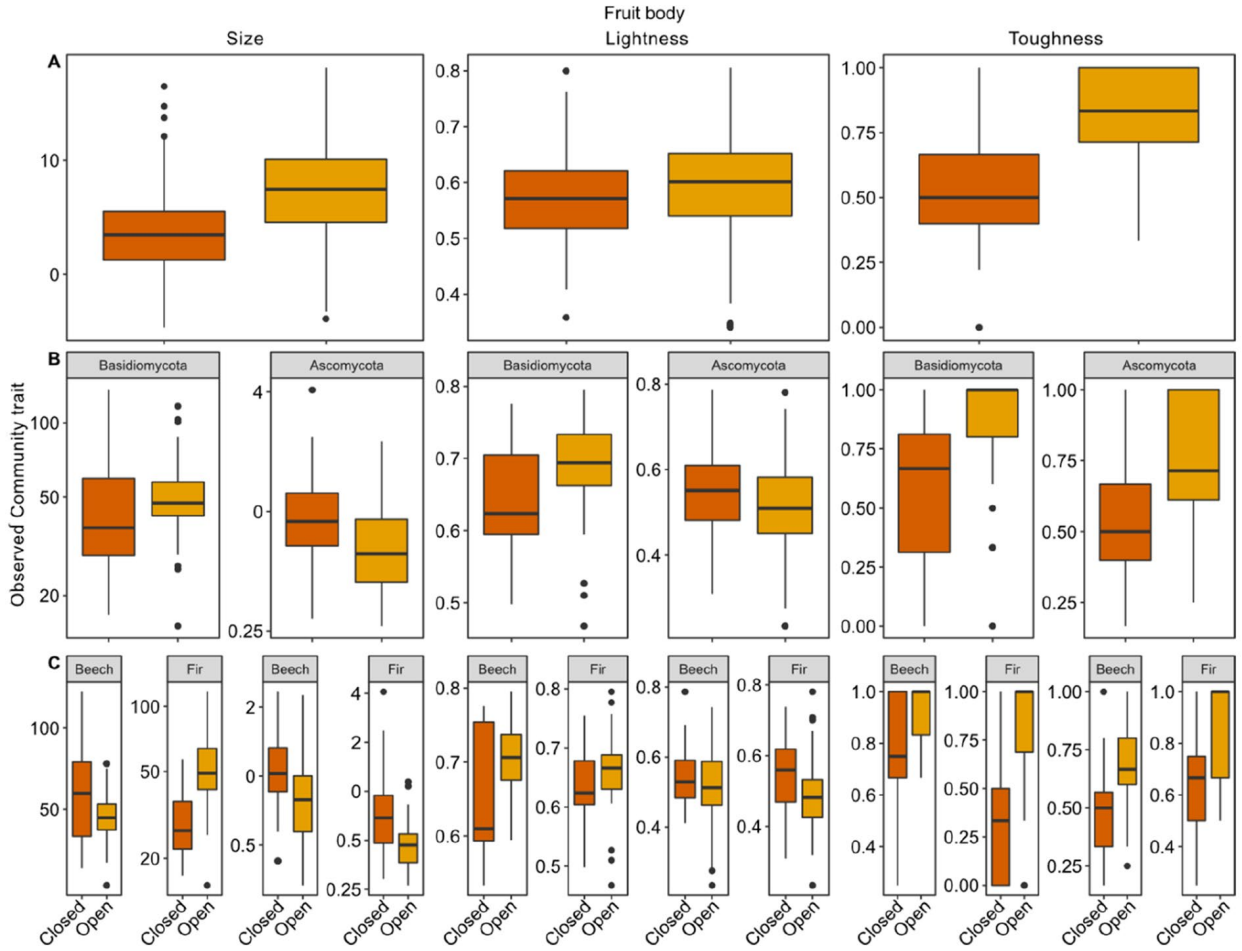
Response of the observed community traits with canopy openness. (**A**) Overall community trait response to canopy openness. Dark orange refers to closed canopies, light orange to open canopies. (**B**) Effects of canopy openness on the community trait separated for Basidio- and Ascomycota. (**C**) Effects of canopy openness on the community trait separated for lineages and tree species. For a statistics table based on a multivariate linear mixed-effects model, see Table 1. Note that fruit body size and color lightness were log_10_-transformed. The unit of fruit body size is millimetres, the unit of lightness and toughness is percentage. R programming environment version 4.1.2^40^ and R add-on package *ggplot2*^41^.

We then tested for responses of the fruiting community traits with canopy openness. In the overall model (across lineages and tree species), we found significantly larger fruit bodies under open canopies (z = 5.79, p < 0.001, Fig. 3; Table 1). Fruit body size, however, differed in response between lineages (Fig. 3; Table 1). Basidiomycota communities showed significantly larger (z = 3.35, p < 0.001), Ascomycota communities significantly smaller fruit bodies under open canopies (z = − 4.62, p < 0.001; Figs. 2, 3; Table 1). Whereas we found opposing effects between the two tree species within Basidiomycota, we found consistent effects within Ascomycota (Fig. 3; Table 1). Fruit body color lightness was significant in only one model. Basidiomycota communities showed significantly lighter fruit bodies under open canopies on beech dead wood (Fig. 3; Table 1). Further, we found a significantly higher proportion of tough-fleshed fruit bodies under open canopies (z = 10.22, p < 0.001; Fig. 3; Table 1), which was significant across lineages and tree species (Fig. 3; Table 1).

**Table 1:** Linear mixed-effects model of the effect of canopy openness on observed community fruit body traits.

	Size (OBS/SES)	Lightness (OBS/SES)	Toughness (OBS/SES)
Intercept	7.41***/− 1.66	− 25.36***/1.16	6.33***/− 4.39***
Canopy openness—open	5.79***/6.45***	− 0.92/− 0.54	10.22***/13.21***
Tree species	− 1.70/− 3.55***	− 0.78/− 1.10	1.60/− 1.25
Size		6.16***/6.09***	0.04/− 0.83
Lightness	6.11***/6.03***		1.06/1.34
Toughness	0.04/− 0.83	0.85/1.13	
R^2^	0.19/0.25	0.15/0.19	0.35/0.34
Basidiomycota–Canopy–Open	3.35***/2.75**	1.35/2.81**	7.94***/9.83***
Basidiomycota–Beech–Canopy–Open	− 1.26/− 2.17*	2.27*/5.17***	2.46*/3.34***
Basidiomycota–Fir–Canopy–Open	5.57***/5.51***	− 0.04/− 0.27	8.38***/10.10***
Ascomycota–Canopy–Open	− 4.62***/− 4.80***	− 1.70/− 1.77	5.92***/6.52***
Ascomycota–Beech–Canopy–Open	− 3.67***/− 3.48***	− 0.90/− 0.98	4.53***/5.72***
Ascomycota–Fir–Canopy–Open	− 3.36***/− 3.68***	− 1.59/− 1.61	4.40**/4.09***

We first tested the effect of the main predictor, canopy openness, on the full community. We then tested the interaction between lineages and canopy; and between lineages, tree species, and canopy. Effect sizes (z values) are displayed for the linear mixed models with a random effect ‘plot in block’. On the left side of each column, the effects of the observed community trait are shown. On the right, the effects for the standardized community trait are based on a null model. Significances are indicated by asterisks, where ‘*’ corresponds to p < 0.05, ‘**’ to p < 0.01 and ‘***’ to p < 0.001. Significant effects were highlighted in bold if both the observed community mean and the standardized effect size (SES) of the community traits were significant. The coefficient of determination is given as marginal R^2^.

**Figure 4:**
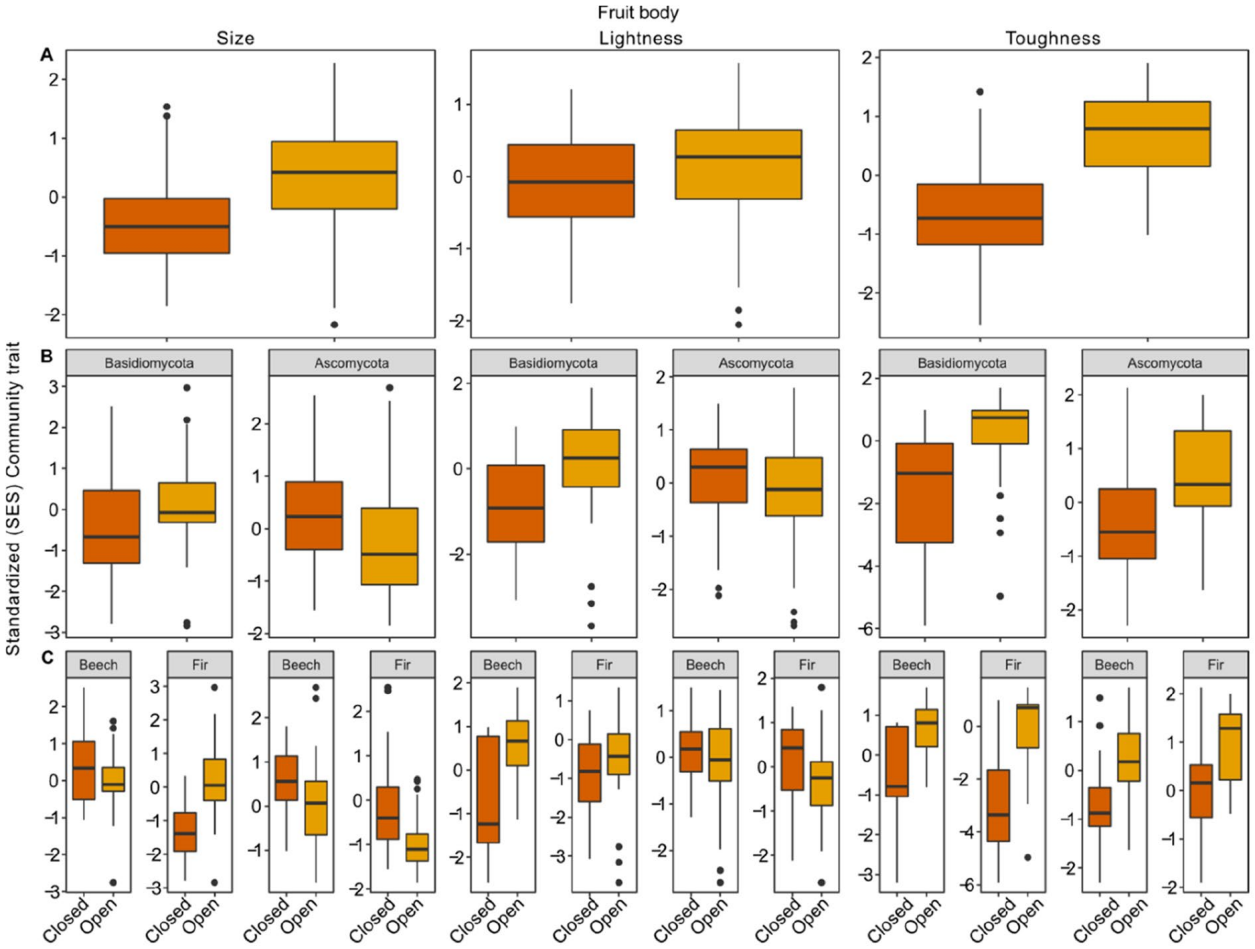
Response of the standardized effect size of the community fruit body traits with canopy openness. Dark orange refers to closed canopies, light orange to open canopies. Standardized effect sizes (SES) were computed based on the independent swap null model. (**A**) Effects of canopy openness on the overall community trait. (**B**) Effects of canopy openness on the community trait separated for Basidio- and Ascomycota. (**C**) Effects of canopy openness on the community trait separated for lineages and tree species. For a statistics table based on a multivariate linear mixed-effects model, see Table 1. R programming environment version 4.1.2^40^ and R add-on package *ggplot2*^41^.

We used a null model to test against a random draw of species in plots. We found consistent responses of the SES community traits of the observed and the standardized community traits (Fig. 4, Table 1).

We further tested the effects of the canopy on all traits in one model using generalized linear models (GLM). The GLM allowed comparing effect sizes between the community traits within one model. The results of the GLMs were largely consistent with the results based on the LMEs (Table S3). Fruit body toughness (z = 7.65) showed the largest relative effect size compared to community fruit body size (z = 5.23) and lightness (z = − 0.41; Table S3).

## Discussion

We found a significant increase in fruiting community toughness with canopy openness and toughness responded most strongly with canopy openness compared with community fruit body size or color lightness. Together, these results suggest that morphological traits of the fruit body at least partly explain previously observed shifts in the fruiting community between microclimates.

Fruit body toughness has not yet been considered directly as a variable. Although toughness was not directly used as a variable in a previous study, soft-fleshed (agaricoid) richness was negative, tough-fleshed (polyporoid) richness of wood-inhabiting fungi was positively affected by forest edges^29^. Like open canopies, forest edges are also characterized by higher sun exposure. Thus, these results are consistent with our findings. Further, an increase in tough-fleshed species was suggested in areas with a pronounced drought season in West Africa^42^. Experimental studies are rare, however, tough-fleshed fruit bodies may provide desiccation protection due to the enhanced stability of the fruit body towards water loss due to either denser hyphal system and thicker cell walls^33^. One experiment found that cell layers with dense cell structures have a lower transpiration rate, but this was tested based on a few species, only^43^. Protection mechanisms against desiccation via a reduction in water loss is a well-known adaption of plants in dry environments. For example, Mediterranean ecosystems are characterized by sclerophyll vegetation with hard leaves^44,45^. More specifically, the olive tree, as a typical Mediterranean plant species, shows strong drought/heat adaptation. Besides lowering water content and water potential in tissues, the olive tree developed morphological adaptations such as thick leaf cuticle and waxy substances, hairiness of the leaf abaxial surface, or high specific weight of leaves^46^. Those adaptations can reduce water loss and thus increase the fitness of the plant^46^. Furthermore, a meta-analysis investigated the dry leaf mass per unit area across species of different biomes. Evergreen species of the Mediterranean Basin, where frequent drought occurs, have the highest dry leaf mass per unit area, resulting from increased thickness. In experiments, dry leaf mass per unit area was higher under elevated light and temperature but also water stress^47^. Thus hard leaves are adaptations of plants towards heat and drought^44,48^. Although such detailed studies are missing for fungal fruit bodies, we carefully suggest (please see limitations below) that fruit body toughness is an adaptation towards heat, drought, and radiation. We thus suggest the toughness-protection hypothesis for macrofungi, stating an advantage of tough-fleshed fruit bodies in hot and dry environments. Finally, we found a stronger effect size of fruit body toughness than fruit body size and color lightness on the microclimatic scale (Table S3). Previous studies showed the relevancy of fruit body size and color on macroclimatic scales^26,27^. Thus, further studies should test if fruit body toughness explains fungal occurrence on macroecological scales. For example, whether biomes associated with hard-leaved plant species also selects for tough-fleshed fungal fruit bodies, e.g., in the Mediterranean biome^44^.

Our results may be carefully interpreted in terms of future climates with more frequent heat and drought periods. Under such conditions, we suggest an increased selection for tough-fleshed fruiting bodies based on our results because they are more likely to be able to endure such conditions. However, whether future climates select for tough-fleshed fruit bodies depends on further considerations. For example, climate change led to a size decline in insect species because maturation is delayed for several reasons, including constrained feeding activities^49^. Similarly, tough-fleshed fruit bodies (e.g., *Fomes* ssp.) require more time to be built up than soft-fleshed fruit bodies (e.g., *Pluteus* ssp.)^50^. Therefore, growth conditions must be suitable over a longer period with more chances of weather extremes interrupting the growth and maturation of the fruit body. Thus tough-fleshed fruit bodies may also be disadvantageous under increasing weather extremes. Therefore, studying fruit body development under harsh conditions from primordium (initial fruit body structure) to maturation (release of spores) of tough- and soft-fleshed species will help understand fungal responses to climate change.

Body size was studied frequently, revealing a body size decline across animals and plants due to climate change^3,49^. Whether fruit body-forming fungi respond with a size decline of mycelium and fruit bodies is unknown. On a global scale, fungal mushrooms communities were smaller in boreal and tropical compared to those in temperate climates^27^. Besides body size, color lightness has been frequently studied in animals and recently also in fungi^26,51^. We expected significant and general responses of fruit body size and color lightness across lineages and hosts, given the prior knowledge based on the existing macroecological studies. We found no consistent community response means of fruit body size and color lightness across lineages or tree species with canopy openness (Fig. 2). However, we found a consistent response of community fruit body size within Ascomycota. Ascomycota communities had smaller fruit bodies under open canopies (Fig. 3, Table 1). Smaller fruit bodies with a higher surface-area to volume ratio get rid of excess heat more rapidly, potentially lowering heat stress under open canopies (‘heat-up-cool-down hypothesis’). Although not significant, Ascomycota communities are generally darker than Basidiomycota communities (Fig. 2), and thus, smaller fruit bodies could potentially counteract overheating via darker fruit bodies, which may counteract damage by radiation (‘photo-protection hypothesis’). Due to the generally small size of most Ascomycota fruit bodies, their fruit bodies develop more closely to the dead-wood surface, which can reach high temperatures under open canopies (Fig. S1). Thus, a rapid release of excess heat may be beneficial for very dark fruit bodies. We found even smaller fruit bodies under open canopies, which would further support this explanation (Fig. 3). Generally, studies on traits in microclimates are scarce. Studies on ant and spider communities found that warmer temperatures under open canopies correlate with larger body size^52–55^. This finding is in contrast to our findings for Ascomycota. One explanation may be that the fruit bodies of most Ascomycota macrofungi are sessile. Insects and spiders, on the other hand, may move in and out of the sunlight to regulate the thermal body condition and thus benefit from better growth conditions while not being affected by harsh conditions.

Finally, our study also contains limitations. First, our data is limited to understanding the full underlying processes shaping fruiting communities. As pointed out in the introduction, the absence of fruit bodies may have other reasons than a maladaptation of the fruit body, e.g., unfavorable mycelial growth conditions. This may have consequences for our inferences and interpretation. Either the fruiting community may be an artefact of the underlying changes in the mycelium community, or even if mycelium communities do not differ between microclimates, fruiting cues may differ (e.g., enhanced damage of the mycelium in harsh microclimate^22^). Differences in fruiting cues may lead to a different subset of the fruiting community. In both cases, differences in the fruiting community between microclimates would not be related to fruit body traits. The species within the fruiting community may differ, however, the trait distribution between microclimates should not differ from a random draw if they are not associated with the microclimate^56,57^. By applying a null model approach, we demonstrated that the response to microclimate differs from a random draw accounting to some extend for uncertainties (Fig. 4, Table 1). Further studies should therefore consider mycelium in their analyses and ideally perform laboratory experiments where microclimate, fruiting cues, and species traits can be manipulated. Second, mycelium or fruiting community changes may not be related to the microclimate but, e.g., associated changes in biotic interactions. Nevertheless, those fungi that produced mature fruit bodies (recorded in our study) should still possess adaptations that allow them to cope with the harsh microclimate. Further, if biotic interactions lead to the observed pattern, this may result from microclimates if our treatment modifies biotic interactions. Thus laboratory studies are required to disentangle independent effects of heat, drought and biotic interactions on fungal mycelium and reproductive growth, e.g., using climate chambers. Third, due to the absence of prior knowledge for most traits in fungal fruit bodies, we restricted our analysis to trait data retrieved from literature (size), photo-measurements (color), and expert knowledge (toughness). The traits were not measured from the specimens which occurred in our study. Thus, the actual traits in situ may differ from those we used. Nonetheless, using average species traits to understand mechanisms of species-environment has considerable value in itself^58^. Ideally, further studies also include intraspecific trait variability^59^ to understand how plastic macrofungi can respond to the microclimate. Fourth, our coding of toughness is based on expert knowledge. Expert knowledge has been demonstrated to be a reliable measure of fruit body traits^60^. One option of directly measuring toughness might be the use of penetration measures, e.g., using a penetrometer. This way, a unique quantification method can be used across species and lineages. However, currently, we are not aware of any study that measured fruit body toughness using quantitative methods.

In conclusion, although trait-based studies in fungi are generally increasing, such efforts are still rare in macrofungi. Our study suggests that differences in the fruiting community between microclimates may be related to differences in fruit body morphology. Morphological traits in harsh microclimates may be relevant to secure fungal reproductive success (fructification until spore release). Climate warming induces more frequent weather extremes and microclimatic harshness^5,61^. Our study suggests that harsh microclimates select species with tough-fleshed fruit bodies in Basidio- and Ascomycota. However, to fully understand how microclimate affects macrofungi, future studies should consider mycelium and reproductive growth (maturation) and sporulation and how morphological traits relate to these processes.

## Material and methods

### Study site and design

This study was part of a larger dead-wood experiment^18^ situated in the management zone of the Bavarian Forest National Park in south-eastern Germany characterized by mixed montane forest, consisting of Norway Spruce (*Picea abies* (L.) H. Karst), European Beech (*Fagus sylvatica* L.) and Silver Fir (*Abies alba* Mill.)^62^. For this study, 120 plots of each 0.1 ha were used in a random block design of five blocks. In autumn 2011, we freshly cut and directly deposited (within less than eight weeks) dead-wood logs (coarse woody debris; mean diameter ± SD = 33 ± 6.5 cm, length = 5 m) of beech and fir. The wood objects were taken from trees of the same age that were harvested from a forest stand of 5 ha of the same elevation. The trees harvested were pairwise, not more distant than 100 m. Further, the soil followed the same homogenous geology (Bohemian Massif, granitic and gneissic bedrock^62^). Each block contained 24 plots, of which half were under open and half under a closed canopy. Further, half of the plots contained a low amount of local dead wood (4 logs of ca. 10 m^3^ ha^−1^), and the other half had a high amount of local dead wood (40 logs of ca. 100 m^3^ ha^−1^). Finally, each block-canopy-amount treatment consisted of two plots with two beech logs, two plots with fir logs, and two plots with one beech and one fir logs. To precisely characterize the amount of dead wood per plot, we calculated the surface area of each object using the formula for a truncated cone. We summed the surface area of all logs and of the sampled objects separately (see below). The surface area was calculated from the length and diameters measured on both ends of each object. To avoid shading by a dense grass layer surrounding the dead-wood logs under open canopies, each plot was mowed once a year during the growing season (for details, see^18^). The open canopies resulted from clearings; an area of 0.1 ha was freed from living or dead trees. Wood surface temperatures were measured on top of 136 logs on a clear summer day, with each five measures per log (half in open, half under closed plots across the four blocks) in August 2018 using an infrared thermal sensor on a summer day^17^. We found differences in temperature between open and closed canopies (Fig. S1).

### Fruit body sampling

We sampled fruit bodies on two logs on each plot for four consecutive years (2012–2015) from a total of 320 sampled objects (hosts). The first year was excluded because records were too few to calculate meaningful community trait means (see “Statistical analysis”). The yearly sampling was during late summer and fall (July–October), the main season of fruit body development^63^. All visible fruit bodies were identified in the field or, if necessary, in the laboratory with the aid of a microscope. Voucher specimens were deposited in the herbarium of the Bavarian Forest National Park. The nomenclature followed MycoBank^64^) and a complete species list is available (Table S1). We considered (i) stipitate- and sessile-pileate Basidiomycota and (ii) Ascomycota with Perithecia or Apothecia. We coded the character values “stipitate- pileate” and “sessile-pileate” based on an published coding dataset of fruit body types for 8400 species^30^. Using this dataset, we used only species which were coded either as “stipitate-pileate” or “sessile-pileate”. We choose to use stipitate- and sessile-pileate as well as Perithecia or Apothecia, to standardize our dataset to fruit body traits where meaningful size measures are available. Based on the fruit body inventories, we produced a presence/absence community matrix. It was recommended that multiple years and surveys are necessary to gain a robust measure of the community^60^, and therefore, we summed occurrences across the sampling years.

### Trait data

We extracted the minimum and maximum width of the fruit body from the literature and public websites (http://www.fungi-without-borders.eu, http://www.pilze-ammersee.de/). Based on the minimum and maximum diameter/width derived from these resources, we calculated the mean fruit body size. In the case of pileate-stipitate Basidiomycota and Apothecia of Ascomycota, we used the diameter of the round-shaped cap. In the case of pileate-sessile Basidiomycota and Perithecia of Ascomycota, we used the width of the fruit body (see Fig. 1 for fruit body types).

We further assembled the color lightness of each species, which is the lightness from the HSL (hue, saturation, lightness) color space model^65^. Therefore, we used a publicly available photograph of each species (mycokey.com, mykoweb.com, 123pilze.de, mushroomobserver.org, mushroomexpert.com, etc.) and sampled nine color pixels from the fruit body photograph, following the protocol developed previously^26^. The protocol entails a quality standard of the photographs, e.g., not over- or underexposed by light, and areas on the surface with dirt or leaves should not be sampled. The nine samples were averaged for each species. This protocol is a reliable approach to estimate fruit body color lightness based on the HSL color space model and was shown to be free from geographic bias and was consistent with color lightness derived from standardized fruit body drawings^26^.

Finally, we attributed each species to either soft- or tough-fleshed (fruit body toughness). Currently, no framework exists to code fruit body toughness across fruit body types and lineages. We thus choose coding based on expert opinion. Consultation of expert knowledge is a robust approach, which has been used to assemble fruit body traits^60^. Based on expert knowledge (among authors and others, see *Acknowledgements*), we first decided to choose two toughness states and then coded all species accordingly. We decided on binary coding, to reflect extremes of toughness. We thus defined tough-fleshed as either hard as wood (e.g., *Fomes* ssp.) or tough (e.g., *Oligoporus* ssp.) as opposed to soft, which was defined as agaricoid softness (e.g., *Agaricus* ssp.). We coded Polyporales, Gloeophyllales, Hymenochaetales as tough-fleshed, and Agaricales as soft-fleshed. This coding roughly reflects the mitic system with agarics having mainly monomitic hyphal system, whereas most Polyporales, Gloeophyllales, and Hymenochaetales have mostly di-, or trimitic hyphal system^66^. The mitic system is not present in Ascomycota. Based on the coding by the authors, fungi with Perithecia were coded as tough-fleshed as opposed to fungi with Apothecia, which were coded as soft-fleshed. Since Basidio- and Ascomycota differ substantially in fruit body size (see above) and toughness, we analysed the datasets for both lineages separately. Please finally note that we were able to retrieve fruit body size for 106, fruit body lightness for 105 and fruit body toughness for 107 species out of 107 species.18/01/2022 21:08:00.

### Statistical analysis

Statistical analyses were conducted in the programming environment R 4.1.2^40^ and images were produced with the add-on R package *ggplot2*^41^.

#### Community data and community trait calculation

To test for differences in fungal fruit body traits between closed and open canopies, we calculated the community trait for each dead-wood log community based on the presence/absence of species. To compute meaningful community traits for each dead-wood log, we used only logs with at least three species. We also considered a threshold of at least one species per log and found consistent results; we further tested a threshold of five species, however, for Basidiomycota on fir, the data was not enough for meaningful estimates. Nonetheless, the overall estimates were consistent with the three species threshold (data not shown).

We computed three response variables. The first response variable was the mean of the log_10_-transformed mean cap diameter across species of each log^28^. The second response variable was the mean of the log_10_-transformed cap color lightness across species of each log. The third response variable was the proportion of tough-fleshed fruit bodies on a log, which was computed as the number of tough-fleshed species divided by the total number of species on a log. Before subjecting the community fruit body toughness to the model, it was arc-sin-transformed to reach a Gaussian distribution. We calculated the community traits for three data subsets: overall considering the full community matrix across lineages; and separately for the lineages: for Basidiomycota and for Ascomycota.

#### Null models

It was previously shown that species richness differs between tree species and canopy openness^18^. Thus, species richness co-varies with the community trait value. Further, the trait pattern observed may also stem from a changed mycelium community, which may have differing fruit body morphology independently of microclimate (artifact scenario). In this case, we would expect a random distribution of traits with microclimate. To account and test for both, an uneven number of species and the artifact scenario, we used a null model (“independent swap”) that randomizes species occurrence across sites but fixes both marginal sums for sites (i.e., species richness of sites) and marginal sums for species (i.e., occupancy of logs across the plots). We first calculated the observed community trait for each log. We then randomized the community data matrix 100 times with the independent swap algorithm^56^ and calculated for each randomized community matrix the community trait. Finally, we calculated the standardized effect size (SES) by subtracting the expected mean (mean across all randomizations) from the observed mean and dividing the difference by the standard deviation across the randomizations for each plot. For the community null model randomization, we used the function *randomizeMatrix* in the R package picante^67^ and used the argument “null.model = ‘independentswap’ “. A significant response of both the observed and SES community trait indicates non-random pattern of community traits in the microclimates^56,68^.

#### Lineage-based trait differences

We were first interested in the general community trait differences between Basidio- and Ascomycota. Generally, different community traits may explain differences in response with our treatment variable (canopy openness). Thus, we used LMEs with the same random effect as above to test the three community traits against lineage separately while using the other traits as covariates (see below).

#### Community trait responses with canopy openness

To assess our hypotheses, we tested the effect of canopy openness on community traits. Testing multiple hypotheses based on the same dataset may lead to unreliable significances (random false positive) due to multiple testing^69^. To account for this issue and test the five hypotheses, we followed three approaches: (1) We tested for covariance among the community trait means and the other covariates. Strong collinearity among co-variables in a model can cause spurious effects and distort models. All pairwise correlation coefficients among the trait variables showed correlation coefficients of |r| < 0.7, a threshold that was recommended not to exceed^70^ to avoid collinearity (Table S2; highest value: 0.66). (2) We fit linear mixed-effects models (LMEs) using the R package *lmer*^71^ and tested the effect of canopy openness on each community trait. In each LME model, we also considered the other two community means as covariates to retain the other variables in the model. (3) Finally, we fit a generalized linear model (GLM, binomial family) with canopy openness as the response variable and fruit body size, color lightness and toughness as predictor variables. This model approach allowed us to integrate all community means in one model to avoid multiple testing in separate models.

Effects of canopy openness on the community traits may be mediated or offset by other factors. Thus, we considered the following variables in the LME and GLM modes. (1) Fungal fruiting communities were shown to significantly differ between tree species^18,72^. For this reason, we included tree species as a binary factor in the overall model and, by using interaction terms, estimated all effects also separately for tree species. (2) Further, the amount of dead wood in the direct proximity of a dead-wood object may affect the stand-microclimate, e.g., with high amount of dead wood capturing and maintaining more moisture than plots with a low amount of dead wood. Thus, we included the sum of surfaces of all logs on a plot in the overall and tree models. The total log surface, however, was not significant in any model. Further, in a previous study testing the effect of this effect on species richness and community composition found no significant effect^18^. Thus, we dropped it from the final models. (3) We used dead-wood logs of the same approximate size, however, the variation may still affect species numbers with slightly larger logs having more species^73^. Thus, to standardize the estimates to the same sampling effort, we additionally added the sampled surface to the models. This variable was significant in no model across observed and standardized community traits. We dropped it from the final models. (4) In the LME models, we further included a random effect, which was ‘plot in block.’

For the LME, we first fit a model with the main fixed effect canopy openness and tree species as further fixed effect covariates (hereafter “overall model”). Note that in this model, we did not include lineage as an effect because the community traits were calculated for the full community matrix. Then we fit a model with an interaction between lineage (Basidio- and Ascomycota), the tree species (Beech and Fir) and canopy openness (closed and open canopy). The interaction was specified by a “*” in the *lmer* function. To test for the pairwise interactions, we used the function *lsmeans* from the R package lsmeans^74^. Using the *lsmeans* function, we first tested for the interaction between lineage and canopy openness to retrieve an estimate of the community trait response with canopy, separate for Basidio- and Ascomycota. Second, we tested the interaction between tree species, lineage and canopy openness to retrieve an estimate of the community trait response with canopy, separate for tree species within lineages. In the GLM we tested for the interactions between lineage, tree species and the predictors using the “:” interaction parameter.

### Ethical approval

The use of plant parts in present study compiles with international, national and/or institutional guidelines. Voucher specimens were deposited in the herbarium of the Bavarian Forest National Park by Lothar Krieglsteiner, Peter Karasch, Andreas Gminder and Frank Dämmrich.

## Supplementary Information

Supplementary Information.

## Data Availability

Data and R code are available via DRYAD (https://doi.org/10.5061/dryad.wh70rxwnx).
