# Supplementary material for: Fungal fruit body assemblages are tougher in harsh microclimates
Source: Sci Rep. 2022 Jan 31;12:1633. doi: 10.1038/s41598-022-05715-9 (PMC8803873; doi:10.1038/s41598-022-05715-9)
Supplement: Supplementary file 1 — Supplementary Information. [file 41598_2022_5715_MOESM1_ESM.pdf]

Fungal fruit body assemblages are tougher in harsh microclimates

Supplementary Material

Supplementary Tables

Table S1 Species list of the species found in this study with values for the fruit body traits.

Phyllum	Species	Fruit body width (mm)	Fruit body lightness	Fruit body toughness	Fruit body type
Ascomycota	*Ascocoryne cylichnium*	12.50	0.69	soft-fleshed	Apotecium
Ascomycota	*Ascocoryne sarcoides*	7.50	0.64	soft-fleshed	Apotecium
Ascomycota	*Ascocoryne solitaria*	17.50	0.46	soft-fleshed	Apotecium
Ascomycota	*Barbatosphaeria barbirostris*	0.40	0.57	tough-fleshed	Peritecium
Ascomycota	*Bertia moriformis*	0.75	0.62	tough-fleshed	Peritecium
Ascomycota	*Bisporella citrina*	2.00	0.48	soft-fleshed	Apotecium
Ascomycota	*Bisporella pallescens*	1.25	0.38	soft-fleshed	Apotecium
Ascomycota	*Calycina discreta*	0.80	0.90	soft-fleshed	Apotecium
Ascomycota	*Calycina languida*	0.35	0.89	soft-fleshed	Apotecium
Ascomycota	*Chaetosphaeria myriocarpa*	0.15	0.49	tough-fleshed	Peritecium
Ascomycota	*Zignoella ovoidea*	0.15	0.19	tough-fleshed	Peritecium
Ascomycota	*Claussenomyces atrovirens*	0.40	0.22	soft-fleshed	Apotecium
Ascomycota	*Coniochaeta ligniaria*	0.50	0.10	tough-fleshed	Peritecium
Ascomycota	*Coniochaeta malacotricha*	0.50	0.48	tough-fleshed	Peritecium
Ascomycota	*Coniochaeta pulveracea*	0.50	0.81	tough-fleshed	Peritecium
Ascomycota	*Coniochaeta subcorticalis*	0.50	NA	tough-fleshed	Peritecium
Ascomycota	*Coniochaeta velutina*	0.50	0.53	tough-fleshed	Peritecium
Ascomycota	*Cosmospora coccinea*	0.30	0.73	tough-fleshed	Peritecium
Ascomycota	*Hypocrea gelatinosa*	2.00	0.80	soft-fleshed	Apotecium
Ascomycota	*Cyathicula cyathoidea*	1.50	0.55	soft-fleshed	Apotecium
Ascomycota	*Exarmidium inclusum*	0.15	0.23	tough-fleshed	Peritecium
Ascomycota	*Haglundia perelegans*	2.00	0.51	soft-fleshed	Apotecium
Ascomycota	*Hamatocanthoscypha laricionis*	0.25	0.42	soft-fleshed	Apotecium
Ascomycota	*Helminthosphaeria stuppea*	0.65	0.18	tough-fleshed	Peritecium
Ascomycota	*Hyaloscypha aureliella*	0.35	0.67	soft-fleshed	Apotecium
Ascomycota	*Hymenoscyphus scutula*	2.75	0.55	soft-fleshed	Apotecium
Ascomycota	*Hymenoscyphus virgultorum*	2.75	0.60	soft-fleshed	Apotecium
Ascomycota	*Lachnellula calyciformis*	2.50	0.83	soft-fleshed	Apotecium
Ascomycota	*Lachnum virgineum*	0.75	0.51	soft-fleshed	Apotecium
Ascomycota	*Lasiosphaeria canescens*	3.00	0.26	tough-fleshed	Peritecium
Ascomycota	*Lasiosphaeria hirsuta*	3.00	0.21	tough-fleshed	Peritecium
Ascomycota	*Lasiosphaeria ovina*	3.00	0.89	tough-fleshed	Peritecium
Ascomycota	*Lasiosphaeria spermoides*	3.00	0.23	tough-fleshed	Peritecium
Ascomycota	*Lasiosphaeria strigosa*	3.00	0.40	tough-fleshed	Peritecium
Ascomycota	*Lophiotrema boreale*	0.70	0.58	tough-fleshed	Peritecium
Ascomycota	*Lophium mytilinum*	0.65	0.84	tough-fleshed	Peritecium
Ascomycota	*Melanomma sanguinarium*	0.40	0.40	tough-fleshed	Peritecium
Ascomycota	*Mollisia aquosa*	0.60	0.91	soft-fleshed	Apotecium
Ascomycota	*Mollisia fusca*	1.10	0.76	soft-fleshed	Apotecium
Ascomycota	*Mollisia ligni*	1.25	0.63	soft-fleshed	Apotecium
Ascomycota	*Mollisia olivaceocinerea*	0.65	0.49	soft-fleshed	Apotecium
Ascomycota	*Natantiella ligneola*	0.40	0.76	tough-fleshed	Peritecium
Ascomycota	*Nectria cinnabarina*	0.25	0.61	tough-fleshed	Peritecium
Ascomycota	*Nectria coccinea*	0.25	0.61	tough-fleshed	Peritecium
Ascomycota	*Dialonectria episphaeria*	0.25	0.50	tough-fleshed	Peritecium

Ascomycota	*Nectria fuckeliana*	0.25	0.46	tough-fleshed	Peritecium
Ascomycota	*Nectria magnusiana*	0.30	0.59	tough-fleshed	Peritecium
Ascomycota	*Nectria peziza*	0.30	0.47	tough-fleshed	Peritecium
Ascomycota	*Ombrophila janthina*	2.50	0.28	soft-fleshed	Apotecium
Ascomycota	*Orbilia coccinella*	0.18	0.65	soft-fleshed	Apotecium
Ascomycota	*Orbilia delicatula*	0.75	0.30	soft-fleshed	Apotecium
Ascomycota	*Patinella sanguineoatra*	1.00	0.22	tough-fleshed	Apotecium
Ascomycota	*Pezicula acericola*	1.25	0.28	soft-fleshed	Apotecium
Ascomycota	*Pezicula cinnamomea*	0.45	0.67	soft-fleshed	Apotecium
Ascomycota	*Phaeohelotium carneum*	0.65	0.26	soft-fleshed	Apotecium
Ascomycota	*Phaeohelotium trabinellum*	0.60	0.28	soft-fleshed	Apotecium
Ascomycota	*Polydesmia pruinosa*	0.50	0.49	soft-fleshed	Apotecium
Ascomycota	*Rosellinia aquila*	1.25	0.74	tough-fleshed	Peritecium
Ascomycota	*Rosellinia thelena*	1.25	0.89	tough-fleshed	Peritecium
Ascomycota	*Scutellinia cejpii*	4.50	0.66	soft-fleshed	Apotecium
Ascomycota	*Scutellinia scutellata*	4.50	0.59	soft-fleshed	Apotecium
Ascomycota	*Scutellinia subhirtella*	5.00	0.63	soft-fleshed	Apotecium
Ascomycota	*Symbiotaphrina microtheca*	0.40	0.75	tough-fleshed	Apotecium
Ascomycota	*Tympanis hypopodia*	0.75	NA	soft-fleshed	Apotecium
Ascomycota	*Tympanis truncatula*	0.75	0.73	soft-fleshed	Apotecium
Basidiomycota	*Bjerkandera adusta*	55.00	0.84	tough-fleshed	pileate-sessile
Basidiomycota	*Clitocybe ditopa*	27.50	0.53	soft-fleshed	pileate-stipitate
Basidiomycota	*Clitopilus hobsonii*	8.50	0.70	soft-fleshed	pileate-stipitate
Basidiomycota	*Coprinus domesticus*	25.00	0.58	soft-fleshed	pileate-stipitate
Basidiomycota	*Coprinellus micaceus*	19.00	0.61	soft-fleshed	pileate-stipitate
Basidiomycota	*Crepidotus cesatii*	18.00	0.39	soft-fleshed	pileate-sessile
Basidiomycota	*Crepidotus pallidus*	1.20	0.65	soft-fleshed	pileate-sessile
Basidiomycota	*Flammulaster carpophilus*	10.00	0.65	soft-fleshed	pileate-stipitate
Basidiomycota	*Gloeophyllum sepiarium*	72.50	0.50	tough-fleshed	pileate-sessile
Basidiomycota	*Gymnopilus sapineus*	42.50	0.56	soft-fleshed	pileate-stipitate
Basidiomycota	*Hohenbuehelia atrocoerulea*	20.00	0.34	soft-fleshed	pileate-sessile
Basidiomycota	*Hohenbuehelia fluxilis*	11.00	0.29	soft-fleshed	pileate-sessile
Basidiomycota	*Hohenbuehelia pinacearum*	7.00	0.39	soft-fleshed	pileate-sessile
Basidiomycota	*Hypholoma capnoides*	40.00	0.56	soft-fleshed	pileate-stipitate
Basidiomycota	*Hypholoma fasciculare*	40.00	0.66	soft-fleshed	pileate-stipitate
Basidiomycota	*Hypholoma marginatum*	30.00	0.48	soft-fleshed	pileate-stipitate
Basidiomycota	*Inonotus hastifer*	NA	0.62	tough-fleshed	pileate-sessile
Basidiomycota	*Inonotus nodulosus*	105.00	0.73	tough-fleshed	pileate-sessile
Basidiomycota	*Irpex lacteus*	400.00	0.73	tough-fleshed	pileate-sessile
Basidiomycota	*Ischnoderma benzoinum*	55.00	0.64	tough-fleshed	pileate-sessile
Basidiomycota	*Laxitextum bicolor*	90.00	0.36	tough-fleshed	pileate-sessile
Basidiomycota	*Mycena amicta*	11.50	0.67	soft-fleshed	pileate-stipitate
Basidiomycota	*Mycena galopus*	15.00	0.65	soft-fleshed	pileate-stipitate
Basidiomycota	*Oudemansiella mucida*	60.00	0.57	soft-fleshed	pileate-stipitate
Basidiomycota	*Panellus mitis*	17.50	0.65	soft-fleshed	pileate-sessile
Basidiomycota	*Panellus serotinus*	65.00	0.63	soft-fleshed	pileate-sessile
Basidiomycota	*Panellus stypticus*	27.50	0.75	soft-fleshed	pileate-sessile
Basidiomycota	*Panellus violaceofulvus*	16.50	0.57	soft-fleshed	pileate-sessile
Basidiomycota	*Pholiota cerifera*	75.00	0.80	soft-fleshed	pileate-stipitate
Basidiomycota	*Pholiota lenta*	75.00	0.75	soft-fleshed	pileate-stipitate
Basidiomycota	*Pholiota tuberculosa*	35.00	0.52	soft-fleshed	pileate-stipitate
Basidiomycota	*Pluteus semibulbosus*	35.00	0.56	soft-fleshed	pileate-stipitate
Basidiomycota	*Polyporus brumalis*	35.00	0.75	tough-fleshed	pileate-stipitate
Basidiomycota	*Postia caesia*	40.00	0.35	tough-fleshed	pileate-sessile
Basidiomycota	*Postia tephroleuca*	35.00	0.48	tough-fleshed	pileate-sessile
Basidiomycota	*Trametes cinnabarina*	65.00	0.59	tough-fleshed	pileate-sessile

Basidiomycota	*Schizophyllum commune*	20.00	0.78	tough-fleshed	pileate-sessile
Basidiomycota	*Simocybe centunculus*	12.50	0.74	soft-fleshed	pileate-stipitate
Basidiomycota	*Trametes gibbosa*	85.00	0.75	tough-fleshed	pileate-sessile
Basidiomycota	*Trametes hirsuta*	57.50	0.76	tough-fleshed	pileate-sessile
Basidiomycota	*Trametes versicolor*	60.00	0.71	tough-fleshed	pileate-sessile
Basidiomycota	*Trichaptum abietinum*	20.00	0.71	tough-fleshed	pileate-sessile

Table S2 Pearson correlation coefficient for the observed community mean of three fruit body traits.

		**Fruit body size**	**Fruit body lightness**	**Fruit body toughness**	**Plot surface (all logs)**
Overall	Fruit body size	–	0.38	0.25	0.05
	Fruit body lightness	0.38	–	0.08	-0.06
	Fruit body toughness	0.25	0.08	–	-0.12
	Plot surface (all logs)	0.05	-0.06	-0.12	–
Basidiomycota	Fruit body size	–	0.18	0.54	0.06
	Fruit body lightness	0.18	–	0.45	0.07
	Fruit body toughness	0.54	0.45	–	-0.03
	Plot surface (all logs)	0.06	0.07	-0.03	–
Ascomycota	Fruit body size	–	0.29	-0.66	0.11
	Fruit body lightness	0.29	–	-0.28	-0.04
	Fruit body toughness	-0.66	-0.28	–	-0.06
	Plot surface (all logs)	0.11	-0.04	-0.06	–

Table S3 Generalized linear model (GLM) with canopy as response variable and the three community traits as predictors, allowing to test the hypotheses family in one model. Residuals were modeled with the binomial family because canopy openness is a binary variable. Significant effects are highlighted in bold. Positive z values correlate with higher community values in the open canopy. Groups were tested using interaction terms with ":" in the GLM.

Group		Predictor	Estimate	Std. Error	z value	Pr(>|z|)
Overall		Intercept	-5.45	1.02	**-5.37**	**<0.000**
		Size	2.51	0.48	**5.23**	**<0.000**
		Lightness	-1.24	3.03	-0.41	0.683
		Toughness	4.84	0.63	**7.65**	**<0.000**
Basidiomycota		Size	0.91	0.86	1.06	0.287
Ascomycota		Size	-0.66	0.90	-0.74	0.460
Basidiomycota		Lightness	2.65	5.22	0.51	0.612
Ascomycota		Lightness	-1.75	1.84	-0.95	0.342
Basidiomycota		Toughness	2.23	0.62	**3.62**	**<0.000**
Ascomycota		Toughness	1.91	0.50	**3.84**	**<0.000**
Basidiomycota	Beech	Size	2.63	1.31	**2.01**	**0.045**
Ascomycota	Beech		-1.48	1.43	-1.04	0.298
Basidiomycota	Fir		0.57	1.20	0.48	0.633
Ascomycota	Fir		-2.94	1.64	-1.80	0.072
Basidiomycota	Beech	Lightness	12.83	8.68	1.48	0.139
Ascomycota	Beech		-1.02	2.49	-0.41	0.682
Basidiomycota	Fir		-3.76	7.05	-0.53	0.593
Ascomycota	Fir		-1.39	2.33	-0.60	0.552
Basidiomycota	Beech	Toughness	1.78	0.89	**2.01**	**0.045**
Ascomycota	Beech		4.54	1.03	**4.42**	**<0.000**
Basidiomycota	Fir		3.16	1.12	**2.81**	**0.005**
Ascomycota	Fir		1.58	0.60	**2.61**	**0.009**

Supplementary Figures

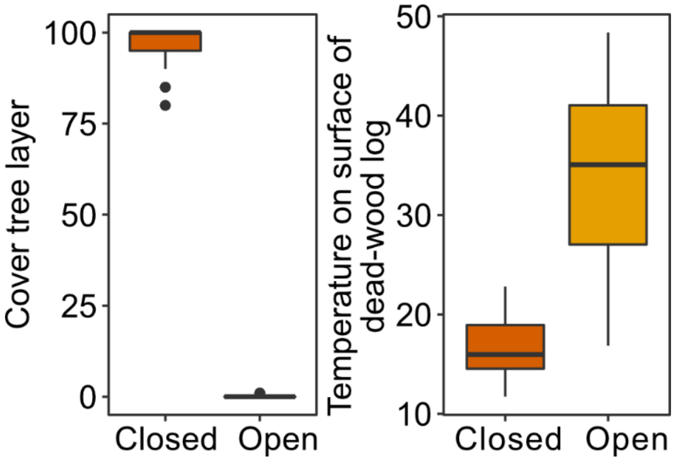

Figure S1 Demonstration of the microclimatic differences between closed (orange) and open canopies (yellow). a) Observational cover of the tree layer. b) Temperature measured on the surface of dead-wood logs on the same sunny day on a subset of the logs in the experiment (5 measures per log. 30 logs in open and 30 logs in closed stand).
